# A systematic review and meta-analysis of selected toxicity endpoints of alpelisib

**DOI:** 10.18632/oncotarget.27770

**Published:** 2020-10-20

**Authors:** Misty Shields, Qianxing Mo, Melissa Armitage, Susan C. Sharpe, Ricardo L.B. Costa

**Affiliations:** ^1^H. Lee Moffitt Cancer Center and Research Institute, Tampa, FL, USA; ^2^Department of Biostatistics, H. Lee Moffitt Cancer Center and Research Institute, Tampa, FL, USA; ^3^Department of Pharmacy, H. Lee Moffitt Cancer Center and Research Institute, Tampa, FL, USA; ^4^Moffitt Biomedical Library, H. Lee Moffitt Cancer Center and Research Institute, Tampa, FL, USA; ^5^Department of Breast Oncology, H. Lee Moffitt Cancer Center and Research Institute, Tampa, FL, USA

**Keywords:** alpelisib, adverse event, hyperglycemia, rash, diarrhea

## Abstract

Purpose: Alpelisib is a first-in-class α-specific phosphatidylinositol 3-kinase inhibitor approved for the treatment of patients with estrogen receptor–positive metastatic breast cancer. High absolute risk (AR) of relevant toxicities has been observed with this treatment. This meta-analysis aimed to improve the precision of the estimated AR of selected adverse events (AEs) associated with this new agent.

Materials and Methods: A literature search was conducted in August 2019 to identify trials analyzing the anti-tumor efficacy and toxicity profile of alpelisib. Heterogeneity was assessed by using *I*^2^ statistics. Data were analyzed using random effect meta-analyses for AR. Eleven trials and 511 patients were included.

Results: There was no evidence of heterogeneity between studies regarding the AR of most AEs except for all-grade weight loss and grade 3–4 stomatitis. The number of serious AEs was clearly reported in only one study, of which the most common was hyperglycemia; the most common all-grade AEs were hyperglycemia (59%), diarrhea (56%), nausea (44%), and rash (38%). Grade 3/4 hyperglycemia and rash occurred in 28% and 10% of patients, respectively. No treatment-associated deaths were observed, and 18% of patients had to stop treatment due to toxicities.

Conclusions: Alpelisib is associated with clinically relevant AEs that can lead to treatment discontinuation. The most common AE was hyperglycemia. No treatment-related deaths were observed.

## INTRODUCTION

Breast cancer is a heterogeneous disease that can be classified according to the presence of transmembrane receptors (i.e., hormonal receptors and human epidermal growth factor receptor 2 [HER2]) as assessed by immunohistochemistry analyses. Most breast cancers are estrogen receptor positive (ER^+^), and treatment with endocrine therapy (ET) improves outcomes among patients with these tumors [1, 2]. Notwithstanding the availability of effective ETs, disease progression is an almost universal challenge for patients with ER^+^ metastatic breast cancer. In this setting, therapies targeting intracellular mechanisms of ET resistance have improved outcomes and have changed clinical practices not only because of improved clinical outcomes but also because of the favorable toxicity profiles of targeted agents. As reviewed by our group, the now widely used CDK4/6 inhibitors are associated with a low absolute risk (AR) of serious adverse events (AEs) and treatment discontinuation rates [3]. Aberrations in the phosphatidylinositol 3-kinase (PI3K)/Akt/mammalian target of rapamycin (mTOR) signaling pathway is a common mechanism of ET resistance [4, 5], and inhibition of this pathway has also improved outcomes among patients with progressive ER^+^ breast cancer. In the pivotal phase 3 trial BOLERO, Baselga et al. showed that an oral mTOR inhibitor (everolimus) improved the median progression-free survival of patients with ER^+^ progressive metastatic breast cancer from 4.1 to 10.6 months (hazard ratio, 0.36; 95% CI, 0.27 to 0.47; *P* < .001) [6]. In May 2019, alpelisib, a novel first-in-class oral small molecule PIK3CA-isoform–specific inhibitor [7], received Food and Drug Administration (FDA) approval for the treatment of metastatic ER^+^ breast cancer harboring *PIK3CA* mutations following progression of disease on or after treatment with ET. This approval was based on the results of the SOLAR-1 trial, in which alpelisib combined with fulvestrant improved the median progression-free survival of patients with this disease to 11.0 months compared with that of 5.7 months in the placebo-fulvestrant group (hazard ratio, 0.65; 95% CI, 0.50 to 0.85; *P* < .001) [8]. Notably, despite its clinical efficacy, treatment with alpelisib was associated with an increased AR of clinically relevant AEs. For example, the AR of serious AEs was 35%, and as much as 25% of patients discontinued treatment with alpelisib due to AEs.

The goal of this systematic review and meta-analysis was to better define the toxicity profile of alpelisib in patients with solid tumors, with particular attention to selected AEs of interest.

## MATERIALS AND METHODS

### Search strategy

A systematic literature search was performed in August 2019 by a medical librarian (S. C. S) across 3 major biomedical literature platforms: PubMed/MEDLINE (1946-), Elsevier’s Embase (1947-), and the Cochrane Library’s Central Register of Controlled Trials (CENTRAL). A combination of free-text keywords, database-specific controlled subject headings, and Cochrane’s Randomized Controlled Trial search filters were used to design the search stratagem for all 3 databases (complete search strategies available in Supplementary Material). Apart from the search hedges, no other filters were applied during the course of the search. All search results were uploaded into Cochrane’s Covidence (https://www.covidence.org/) for deduplication, screening, reference management, and data extraction.

### Selection of trials and data extraction

Our analysis included clinical trials of any phase in which alpelisib was used in nonpediatric populations and studies assessing efficacy or safety of alpelisib in combination with other treatments. Clinical trials were selected by the primary reviewer (R. L. B. C) and independently reviewed by 1 secondary reviewer (M. S.). The primary reviewer (R. L. B. C) made all final decision in cases of assessment discordance.

From included trials, we extracted the total number of patients evaluable for toxicity, the number of all grade AEs, the number of grades 3–4 toxicities, the number of deaths, and the number of patients who discontinued treatment because of treatment-related AE. Furthermore, we documented the number of selected all-grade AEs (i.e., fatigue, nausea, diarrhea, hyperglycemia, rash, alopecia, weight loss, decreased appetite, asthenia, stomatitis, mucosal inflammation, dysgeusia, and arthralgia). These AEs were selected because they were the most commonly observed AEs associated with PI3K/AKT/mTOR-targeted therapies or because of their clinical relevance. The relationship between AEs and treatment administration (i.e., treatment-related AEs vs all causality) was also documented when these data were available. From phase 1 dose-escalation studies, we extracted the number of toxicity events from the cohorts treated with the dose-regimen closest to the FDA-approved alpelisib dose-regimen (i.e., 300 mg daily on 28-day cycles).

### Statistical methods

To obtain the pooled fixed-effect and random-effect estimates, we conducted meta-analyses of one-sample proportions using meta package in R 3.6.1 (https://www.r-project.org/). Heterogeneity was assessed via the *I*^2^ statistic, and the presence of study heterogeneity was examined using the *Q*-test. Finally, publication bias was evaluated via Egger’s test.

## RESULTS

### Included and excluded studies

**Figure 1:**
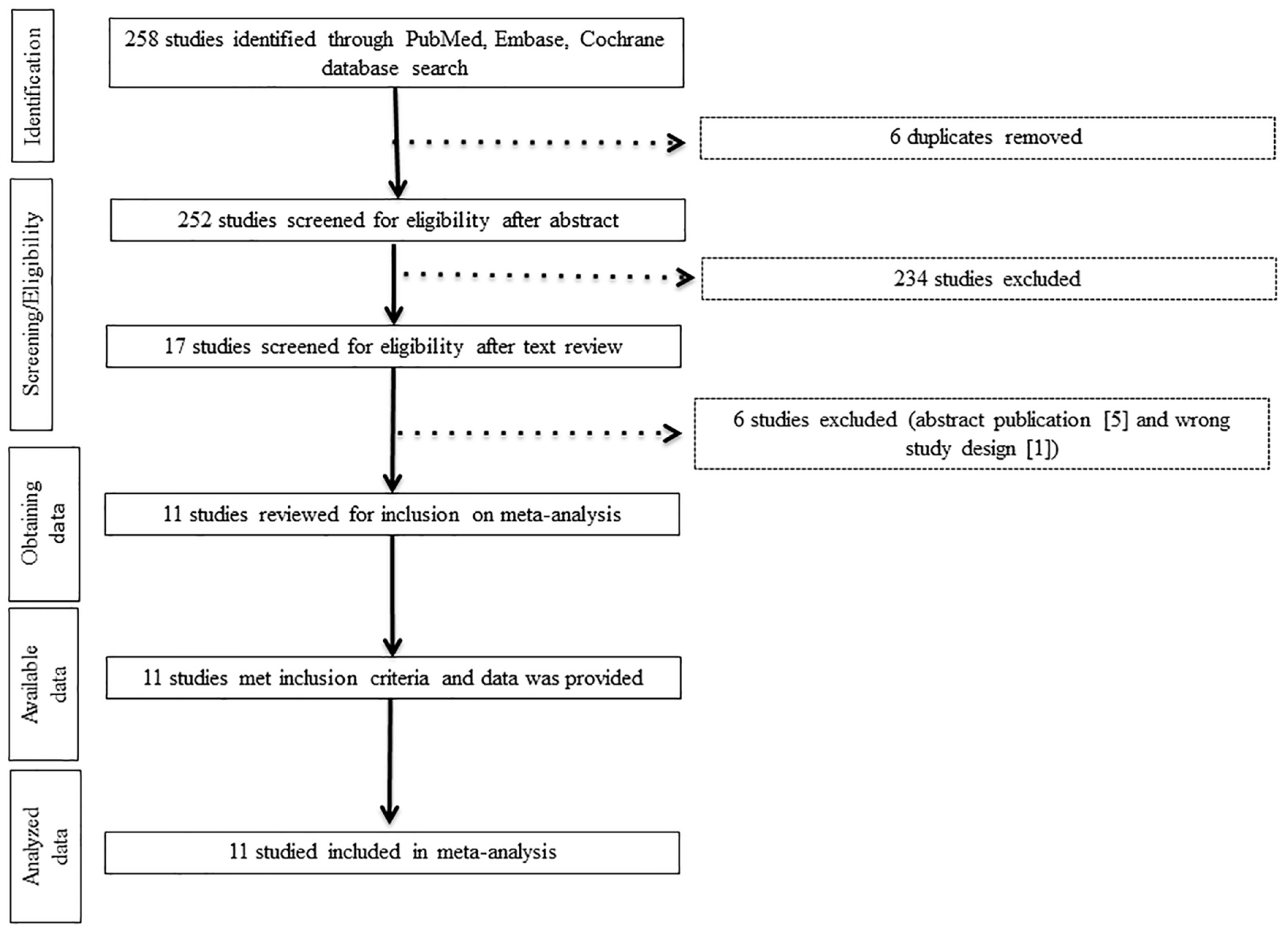
Study flow diagram. This double-column figure was created using PowerPoint. Abbreviations: FDA, Food and Drug Administration.

Our librarian-guided literature search yielded 258 studies with 6 duplicates obtained through PubMed, EMBASE, and Cochrane. A total of 234 studies were excluded during the title and abstract screening. After text review, 7 additional studies were excluded for not meeting the inclusion criteria (Figure 1). Eleven studies met the inclusion criteria, and data from them were extracted (Table 1). These studies included one phase 3, one phase 2, and nine phase 1 trials. None of the 11 trials reported the median follow-up time in their data presentation. Only 2 studies [9, 10] clearly reported AEs suspected to be treatment related (See Table 1).

**Table 1:** Clinical trial publications included in the meta-analysis^a^

PubMed ID (year of publication)	https://clinicaltrials.gov ID	First author	Phase	Type of cancer	Trial clearly excluded patients with DM	No. patients evaluable for toxicity	Tumor stage	Combination	No. patients with ECOG PS ≥ 2	Median Age, y	NCI CTCAEs Scale
31091374 (2019) [8]	NCT02437318	Andre F	3	Breast cancer	No	284	Metastatic	Fulvestrant	0	63	4.03
27126994 (2017) [13]	NCT01791478	Mayer I	1	Breast Cancer	Yes	20^b^	Metastatic	Letrozole	0	53	4
30543347 (2019) [9]	NCT01219699	Juric D	1	Breast Cancer	No	9^b^	Metastatic	Fulvestrant	0	NR	4
29401002 (2018) [14]	NCT01219699	Juric D	1	Multiple histology	Yes	23^b^	Metastatic	None	6	59	4
30588709 (2019) [15]	NCT01387321	Ando Y	1	Multiple histology	Yes	14^b^	Metastatic	None	1	NR	4
29850984 (2018) [16]	NCT02038010	Jain S	1	Breast cancer	Yes	6^b^	Metastatic	T-DM1	NR	NR	4.03
30880072 (2019) [17]	NCT01623349	Konstantinopoulos P	1	Multiple histology	No	6^b^	Metastatic	Olaparib	0	NR	4.03
30723140 (2019) [18]	NCT01923168	Mayer I	2	Breast cancer	No	130	Nonmetastatic	Letrozole	0	65.5	4.03
30167089 (2018) [10]	NCT02051751	Rodon J	1	Multiple histology	No	6^b^	Metastatic	Paclitaxel	0	NR	4.03
28363909 (2017) [19]	NCT01719380	Van Geel RMJM	1	Colorectal cancer	No	10	Metastatic	Encorafenib and Cetuximab	0	NR	4
31678634 (2019) [20]	NCT02282371	Dunn LA	1	Head and Neck cancer	Yes	3^b^	Metastatic and nonmetastatic	Cetuximab and radiation therapy	0	NR	4

^a^The alpelisib dose regimen for all trials, except for NCT01387321, was 300 mg daily. NCT01387321 was an early-phase trial that did not include a 300 mg dose regimen cohort; the dose regimen for this study was 350 mg daily. ^b^These represent the number of patients treated at 300 mg daily dose regimen in dose-escalation trials. Abbreviations: DM, diabetes mellitus; ECOG PS, Eastern Cooperative Oncology Group performance status; NCI CTCAEs, National Cancer Institute Clinical Trial Criteria Adverse Events; NR, not reported; T-DMI1, trastuzumab emtansine (T-DM1).

### Description of study participants

In the 11 included studies, a total of 511 patients were evaluable for toxicity. Six of the patients had ECOG performance status of 2, and the remainder had a performance status of 0 to 1. The median age of study participants was only reported in 3 studies (range, 53–62 years). Four of these trials enrolled 319 evaluable for toxicity patients with metastatic breast cancer; out of all 11 trials, there was 1 trial that enrolled patients with nonmetastatic breast cancer disease; 130 patients were evaluable for toxicity. The remainder of studies enrolled patients with other solid tumors (Table 1).

### Study-to-study heterogeneity and publication bias

*I*^2^ statistics revealed that interstudy heterogeneity was 79% for all-grade weight loss (*P* < .01) and 90% for grade 3–4 stomatitis (*P* = .04). The results of heterogeneity testing between studies for selected all-grade and grade 3/4 AEs is presented in Tables 2 and 3. Testing for publication bias did not show significant results (See Supplementary Material).

**Table 2:** AR of selected all-grade AEs

All-grade AE	Fixed-effect AR, % (95% CI range)	Random-effect AR, % (95% CI range)	*I*^2^, %^a^	*P* value
Alopecia	0.20 (0.17; 0.24)	0.20 (0.17; 0.24)	0	0.88
Fatigue	0.31 (0.27; 0.35)	0.34 (0.26; 0.43)	52	0.01
Asthenia	0.19 (0.16; 0.23)	0.19 (0.16; 0.23)	0	0.54
Nausea	0.44 (0.40; 0.49)	0.44 (0.40; 0.49)	0	0.25
Vomiting	0.24 (0.20; 0.28)	0.23 (0.17; 0.29)	16	0.5
Diarrhea	0.56 (0.52; 0.60)	0.56 (0.52; 0.60)	0	0.08
Weight loss	0.22 (0.18; 0.26)	0.19 (0.09; 0.35)	79	< 0.01
Decreased appetite	0.34 (0.30; 0.38)	0.34 (0.30; 0.38)	0	0.71
Stomatitis	0.27 (0.23; 0.32)	0.28 (0.23; 0.33)	10	0.45
Mucosal inflammation	0.18 (0.14; 0.22)	0.18 (0.14; 0.22)	0	0.36
Dysgeusia	0.18 (0.15; 0.22)	0.18 (0.15; 0.22)	0	0.24
Rash	0.38 (0.33; 0.42)	0.38 (0.33; 0.43)	7	0.53
Pruritus	0.18 (0.14; 0.21)	0.18 (0.14; 0.21)	0	0.73
Hyperglycemia	0.60 (0.56; 0.64)	0.59 (0.51; 0.66)	37	0.04
Arthralgia	0.12 (0.08; 0.16)	0.12 (0.08; 0.16)	0	0.32

Abbreviations: AEs, Adverse Events; AR, Absolute Risk; CI, Confidence Interval. ^a^Percentage of variance between studies that is due to heterogeneity and not chance.

**Table 3:** AR of selected grade 3-4 AEs

Grade ≥ 3 AEs	Fixed-effect AR, % (95% CI range)	Random-effect AR, % (95% CI range)	*I*^2^,%^a^	*P* value
Fatigue	0.03 (0.01; 0.04)	0.03 (0.01; 0.04)	0	0.95
Asthenia	0.01 (0.00; 0.03)	0.01 (0.00; 0.05)	3	1
Nausea	0.02 (0.01; 0.04)	0.02 (0.01; 0.04)	0	1
Vomiting	0.01 (0; 0.002)	0.01 (0; 0.002)	0	0.94
Diarrhea	0.05 (0.03; 0.08)	0.04 (0.02; 0.09)	19	0.58
Weight loss	0.04 (0.02; 0.06)	0.04 (0.02; 0.06)	0	1
Decreased appetite	0.00 (0; 0.02)	0.00 (0; 0.02)	0	1
Stomatitis	0.02 (0.01; 0.04)	0.01 (0.00; 0.30)	90	0.04
Mucosal inflammation	0.02 (0.01; 0.04)	0.02 (0.01; 0.04)	0	1
Rash	0.10 (0.08; 0.13)	0.10 (0.08; 0.13)	0	0.84
Pruritus	0.01 (0; 0.02)	0.01 (0; 0.02)	0	1
Hyperglycemia	0.32 (0.28; 0.36)	0.28 (0.21; 0.37)	36	0.13
Arthralgia	0 (0; 0.32)	0 (0; 0.32)	0	1

^*^Percentage of variance between studies that is due to heterogeneity and not chance. Abbreviations: AEs, Adverse Events; AR, Absolute Risk; CI, Confidence Interval.

### Absolute risk of serious AEs, death, and number of patients who discontinued treatment due to toxicity

**Figure 2:**
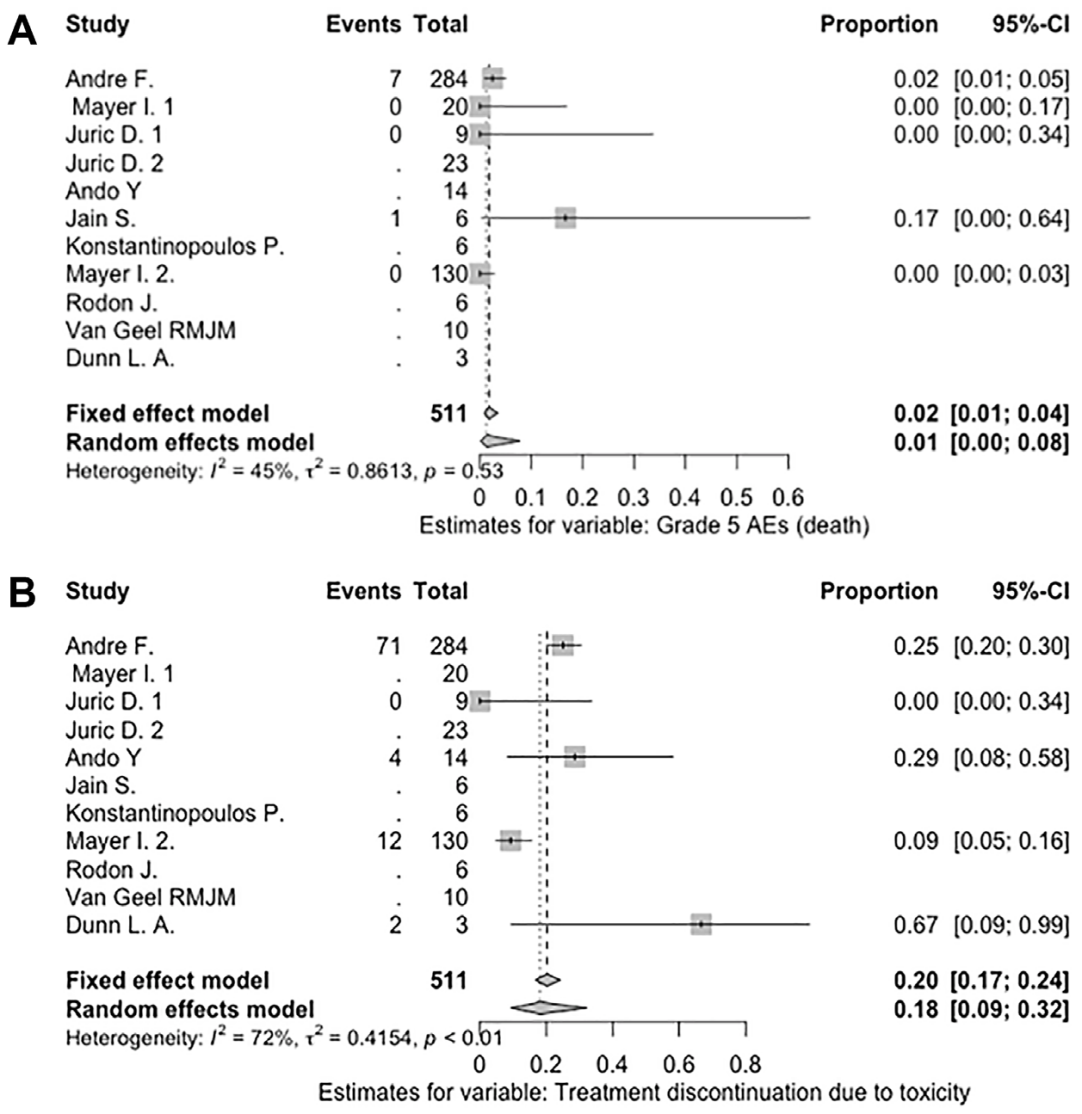
Pooled AR of death and discontinuation of treatment due to toxicity. This double-column figure was created using the meta package in R 3.6.1. (**A**) Pooled AR of death. (**B**) Pooled AR of treatment discontinuation due to toxicity. Abbreviations: AR, absolute risk; CI, confidence interval.

The number of serious AEs was reported in only 1 trial [8], which noted that 99 out of 284 study participants experienced a serious AE (AR = 34.9%). AR for serious AEs was not pooled, as the other studies did not report the number of serious AEs. The most common serious AEs were hyperglycemia (10%), followed by diarrhea (3%), abdominal pain (2%), and acute kidney injury (2%), which were reported regardless of relation to treatment. The pooled random effect of AR of death was 1%, but none of the 8 deaths were clearly associated with alpelisib. Six patients died from cancer progression, 1 died from a second primary cancer progression, and 1 died from cardiopulmonary arrest (not specified). The pooled random effect AR of treatment discontinuation due to toxicity was 18%. Notably, significant heterogeneity was observed *I*^2^ = 72% (*P* < .01) (see Figure 2).

### Absolute risk of selected AEs

The most common all-grade, all-causality AEs were hyperglycemia, with a random-effect AR of 59% (95% CI, 0.51–0.66), followed by diarrhea (56%), nausea (44%), rash (38%), decreased appetite (34%), and fatigue (34%) (Table 2). Despite the number of all-grade AEs that could lead to weight loss (eg, nausea, diarrhea, and stomatitis), the AR of all-grade (19% [95% CI 0.09–0.35]) and grade ≥ 3 (4% [95% CI 0.02–0.06]) weight loss was observed in a small number of patients. All grade 3–4 AEs were observed in < 10% of patients, except for hyperglycemia (AR 28% [95% CI, 0.21–0.37]) and rash (AR 10% [95% CI, 0.08–0.13]) (Table 3). No grade 3–4 dysgeusia was observed, which is why the AR was not pooled.

## DISCUSSION

In recent years, targeted therapies have improved the overall survival rates of patients with ER^+^ and HER2^-^ metastatic breast cancer [11, 12]. For instance, CDK4/6 inhibitors have become standard treatments for these patients, as these agents significantly improve clinical outcomes and carry a low AR of serious AEs. In 2012, results from the pivotal BOLERO trial confirmed that inhibition of PI3K/AKT/mTOR signaling pathway by everolimus leads to clinically meaningful improvement in outcomes [6]. In parallel, this oral mTOR inhibitor has been associated with significant AR of clinically relevant AEs, and treatment with lower-dose regimens of this drug has been used to allow for treatment continuation. Alpelisib is a first-in-class agent approved for the treatment of patients with ER^+^ and HER2^-^ metastatic breast cancer harboring *PIK3CA* mutations [8]. Our systematic review summarized the available data on the toxicity profile of this new agent.

The majority of the patients included in this study had a diagnosis of breast cancer and were treated at the FDA-approved dose of alpelisib of 300 mg daily by mouth (see Table 1), thus strengthening the external validity of the results presented. There was no significant heterogeneity among the majority of ARs pooled, which likely suggests that most AEs reported in the 11 trials analyzed were treatment related. The most common all-grade AE was hyperglycemia (AR of 59%), and 28% of cases were grade 3/4 AEs with only one case of ketoacidosis found in our literature search (SOLAR-1 study). Hyperglycemia was also the most common serious AE. Remarkably, the trials in our analysis either excluded patients with a history of diabetes or mandated the documentation of an adequate glucose control at study entry (ie, fasting plasma glucose ≤ 140 mg/dL and glycosylated hemoglobin ≤ 6.4%). The obvious corollary is that treating physicians should be aware of this AE. As per the directions of the package insert, patients’ fasting plasma glucose and glycosylated hemoglobin levels should be closely and routinely monitored while on alpelisib, given the high AR of hyperglycemia, and treated promptly with oral anti-diabetic agents. Interestingly, other PI3K inhibitors have been associated with a toxicity profile similar to that of alpelisib, including an increased risk of hyperglycemia [11, 12]. Rash is also a possible complication from alpelisib and treatment with topical corticosteroids and oral antihistamine should be considered early on. Other clinically relevant AEs that were also observed included diarrhea, nausea, and vomiting; aside from hyperglycemia and rash, the AR of these grade 3–4 AEs was ≤ 10% (Table 3). Noticeably, none of the deaths reported in the 11 trials reviewed were related to alpelisib. One limitation of our meta-analysis is that we did not have access to individual patient data, which limited our ability to explore correlations between patient characteristics and the AR of AEs. Nonetheless as the authors did not report the median follow up time of study populations the time to initiation of AEs could not assessed. Given the ongoing development of clinical trials with alpelisib in the treatment of solid tumors (eg, NCT04208178, NCT04216472, NCT03386162, NCT03631953), strategies to diagnose and mitigate common AEs are needed.

In summary, treatment with alpelisib is associated with clinically relevant AEs, which, despite being closely monitored in clinical trials, have led to treatment discontinuation in as much as 20% of patients. Hyperglycemia is the most common AE associated with this novel agent. Patients treated with alpelisib require close monitoring, early diagnosis, and management of AEs.

## SUPPLEMENTARY MATERIALS




