# Supplementary material for: A systematic review and meta-analysis of selected toxicity endpoints of alpelisib
Source: Oncotarget. 2020 Oct 20;11(42):3793–9. doi: 10.18632/oncotarget.27770 (PMC7584237; doi:10.18632/oncotarget.27770)
Supplement: Supplementary file 1 [file oncotarget-11-3793-s001.pdf]

A systematic review and meta-analysis of selected toxicity endpoints of alpelisib

SUPPLEMENTARY MATERIALS

R Markdown

The following forest plots show the meta analysis results for variables of interest pertaining to selected toxicities of an oral PIK3CA inhibitor (alpelisib). Both fixed effects and random effects models were considered to be a test for heterogeneity for each toxicity. Analyses were run in R 3.6.1. See Supplementary Materials.

```
## Loading 'meta' package (version 4.9-9).
## Type 'help(meta)' for a brief overview.
```
