# Supplementary material for: A systematic review and meta-analysis of selected toxicity endpoints of alpelisib
Source: Oncotarget. 2020 Oct 20;11(42):3793–9. doi: 10.18632/oncotarget.27770 (PMC7584237; doi:10.18632/oncotarget.27770)
Supplement: Supplementary file 2 [file oncotarget-11-3793-s002.docx]

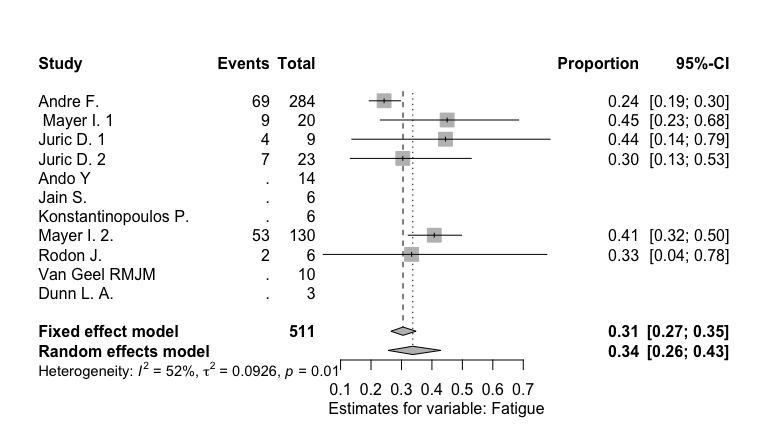

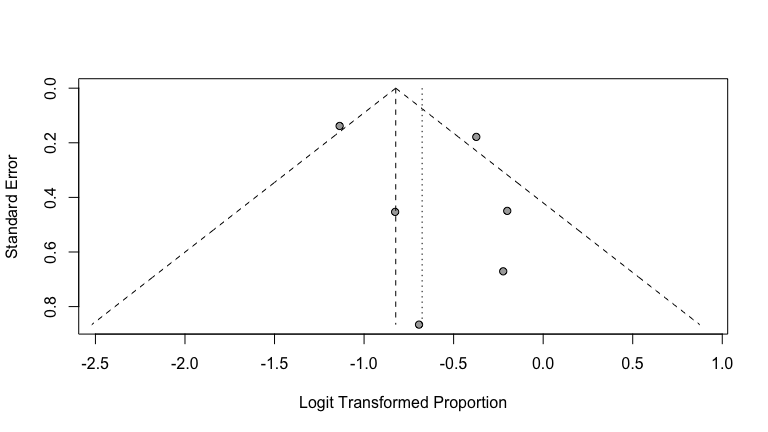


## Egger’s test
## Linear regression test of funnel plot asymmetry
##
## data: m1
## t = 0.93265, df = 4, p-value = 0.4038
## alternative hypothesis: asymmetry in funnel plot
## sample estimates:
## bias se.bias slope
## 1.149444 1.232453 -1.035165


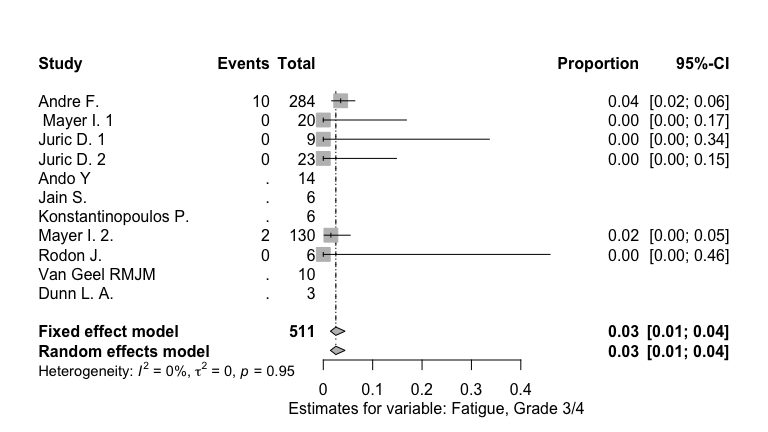

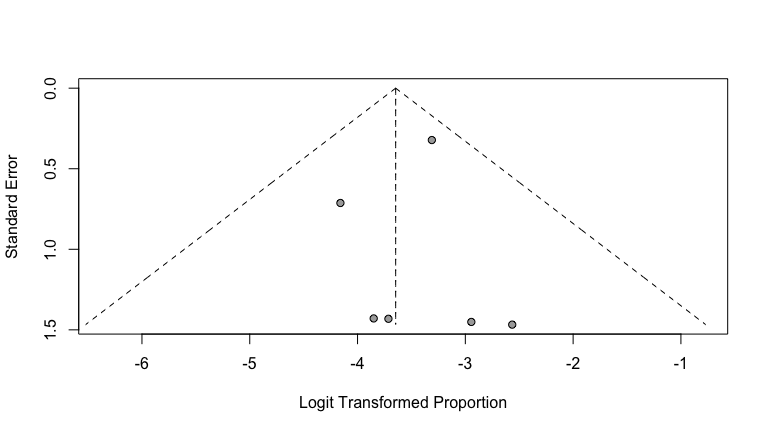


## Egger’s test
## Linear regression test of funnel plot asymmetry
##
## data: m2
## t = -0.23341, df = 4, p-value = 0.8269
## alternative hypothesis: asymmetry in funnel plot
## sample estimates:
## bias se.bias slope
## -0.1067504 0.4573481 -3.3721979


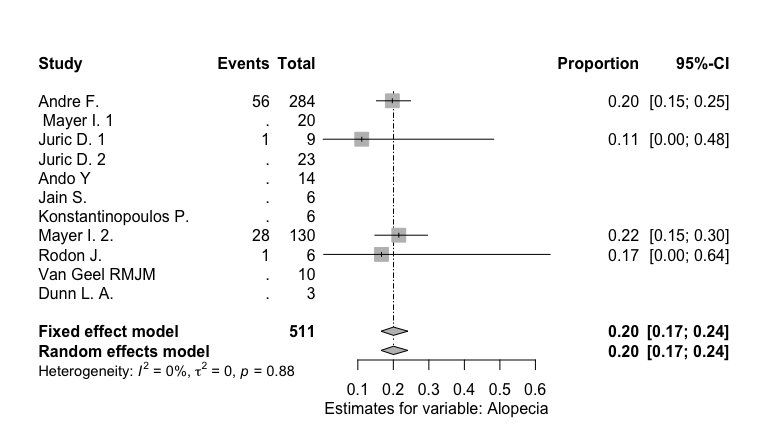

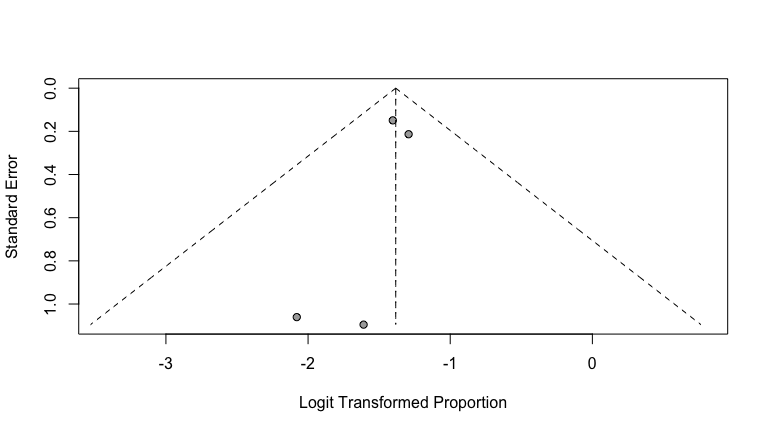


## Egger’s test
## Linear regression test of funnel plot asymmetry
##
## data: m3
## t = -1.1637, df = 2, p-value = 0.3646
## alternative hypothesis: asymmetry in funnel plot
## sample estimates:
## bias se.bias slope
## -0.4334670 0.3724955 -1.2959890


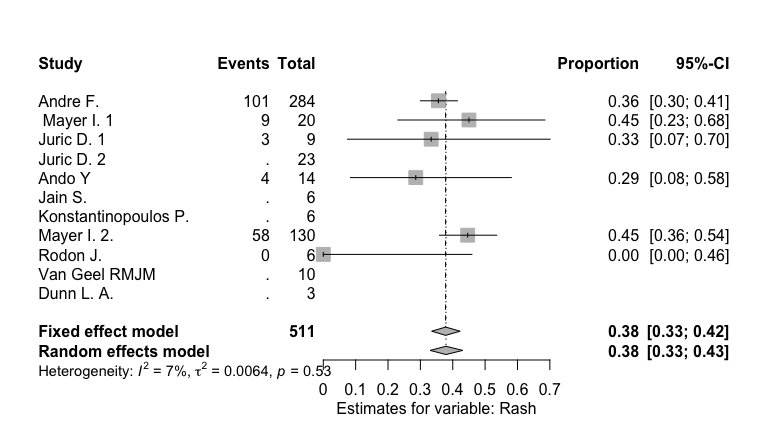

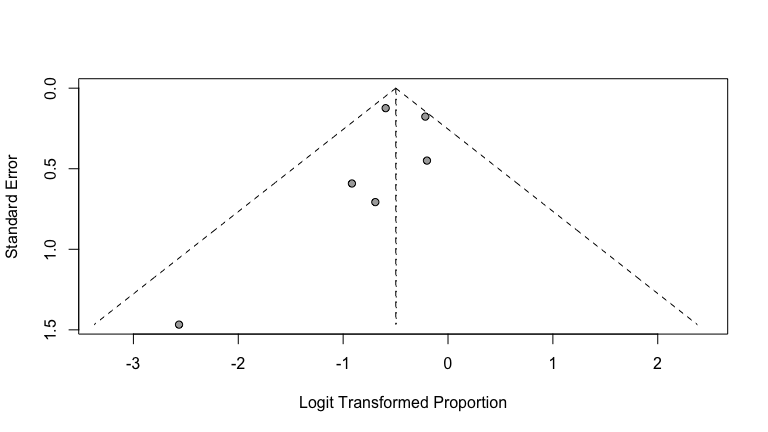


## Egger’s test
## Linear regression test of funnel plot asymmetry
##
## data: m5
## t = -0.67802, df = 4, p-value = 0.535
## alternative hypothesis: asymmetry in funnel plot
## sample estimates:
## bias se.bias slope
## -0.5158948 0.7608885 -0.3873383


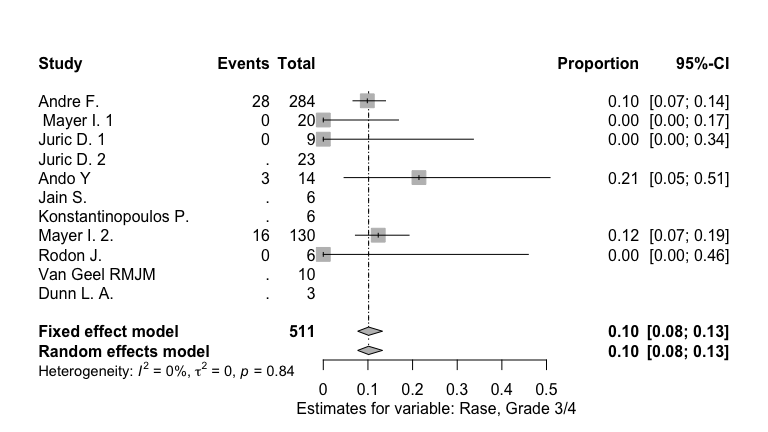

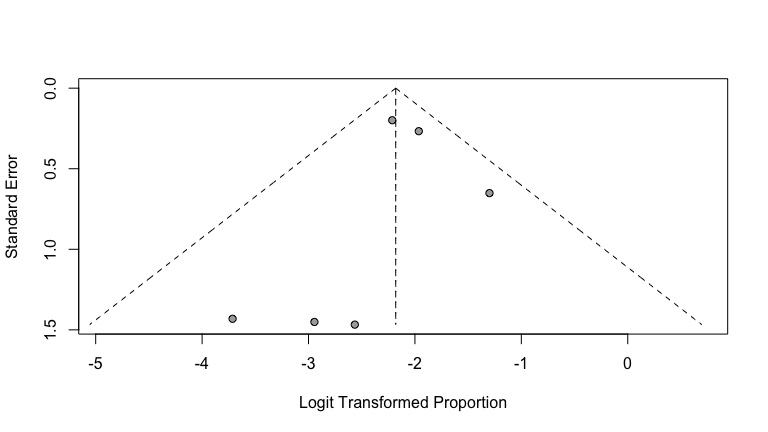


## Egger’s test
## Linear regression test of funnel plot asymmetry
##
## data: m6
## t = -0.4883, df = 4, p-value = 0.6509
## alternative hypothesis: asymmetry in funnel plot
## sample estimates:
## bias se.bias slope
## -0.2957570 0.6056837 -2.0255625


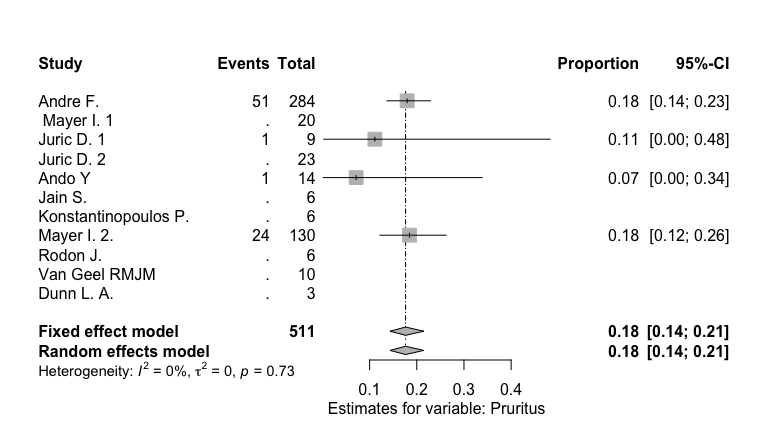

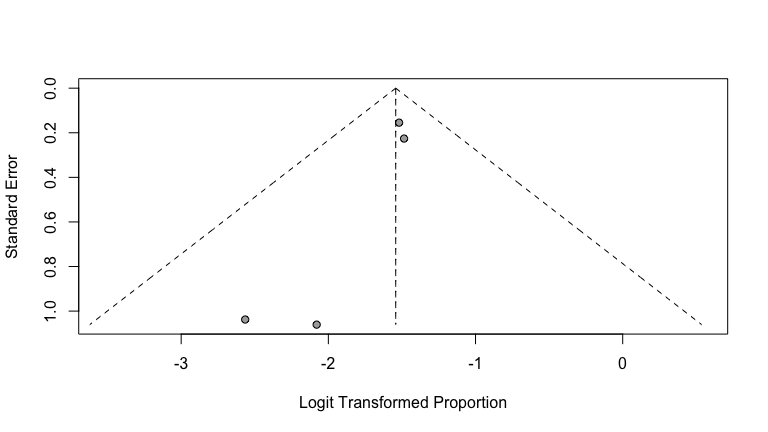


## Egger’s test
## Linear regression test of funnel plot asymmetry
##
## data: m7
## t = -2.9407, df = 2, p-value = 0.0988
## alternative hypothesis: asymmetry in funnel plot
## sample estimates:
## bias se.bias slope
## -0.8677225 0.2950714 -1.3563325


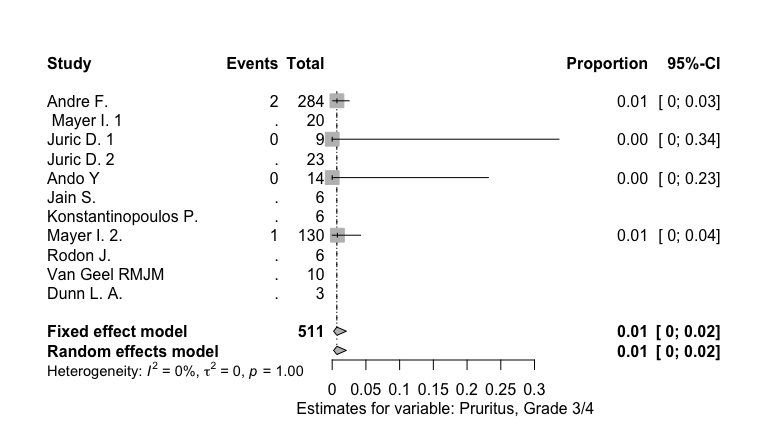

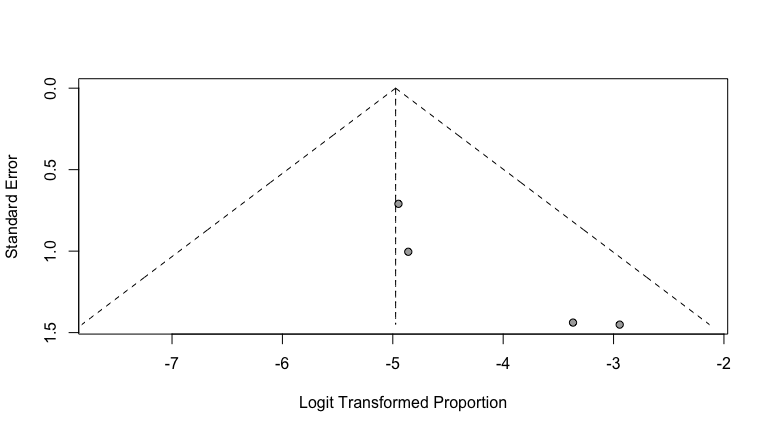


## Egger’s test
## Linear regression test of funnel plot asymmetry
##
## data: m8
## t = 3.4658, df = 2, p-value = 0.07412
## alternative hypothesis: asymmetry in funnel plot
## sample estimates:
## bias se.bias slope
## 2.367177 0.683012 -6.769241


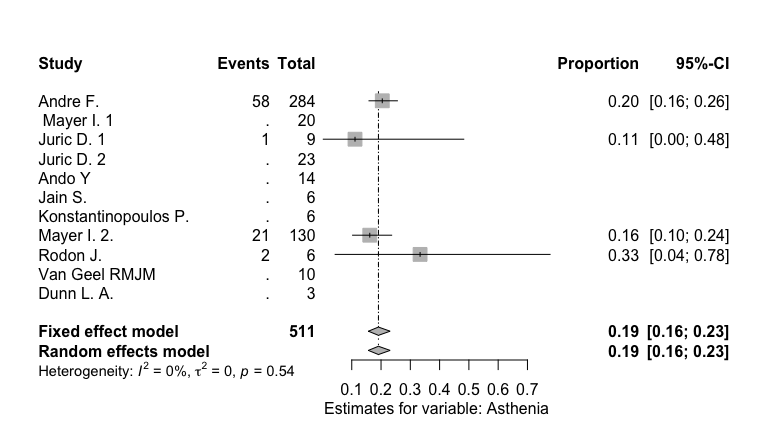

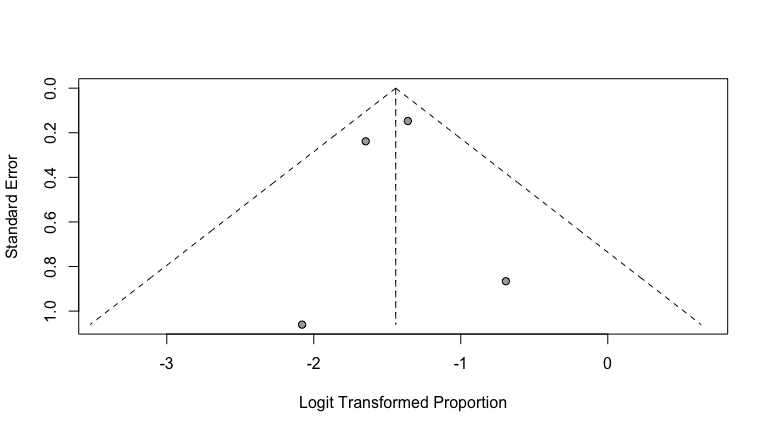


## Egger’s test
## Linear regression test of funnel plot asymmetry
##
## data: m9
## t = -0.13028, df = 2, p-value = 0.9083
## alternative hypothesis: asymmetry in funnel plot
## sample estimates:
## bias se.bias slope
## -0.1135538 0.8715990 -1.4102967


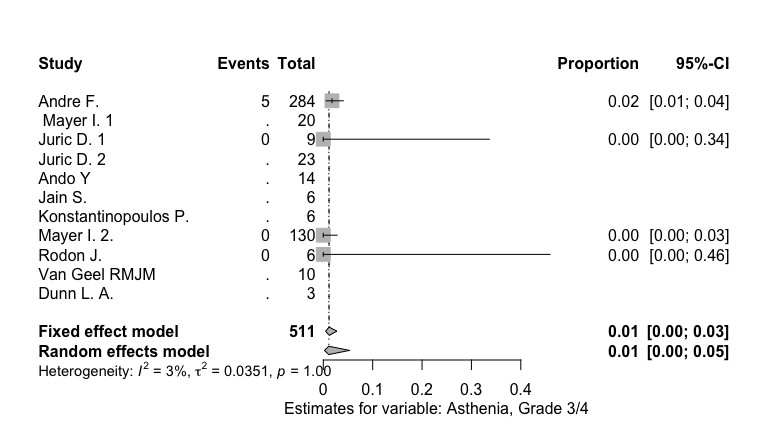

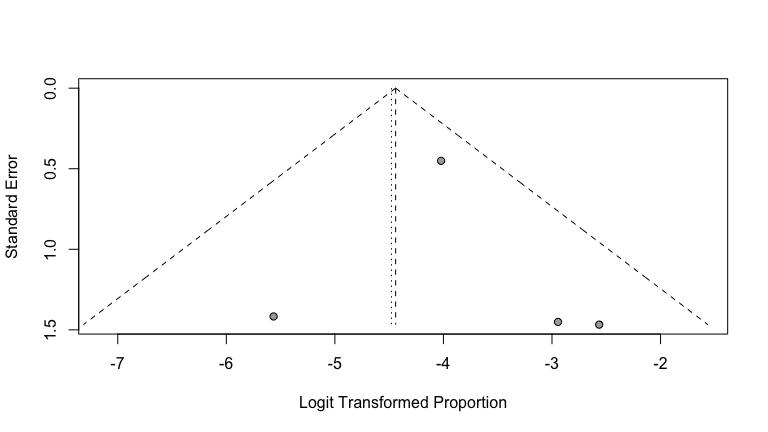


## Egger’s test
## Linear regression test of funnel plot asymmetry
##
## data: m10
## t = 0.30502, df = 2, p-value = 0.7892
## alternative hypothesis: asymmetry in funnel plot
## sample estimates:
## bias se.bias slope
## 0.3302974 1.0828625 -4.1789805


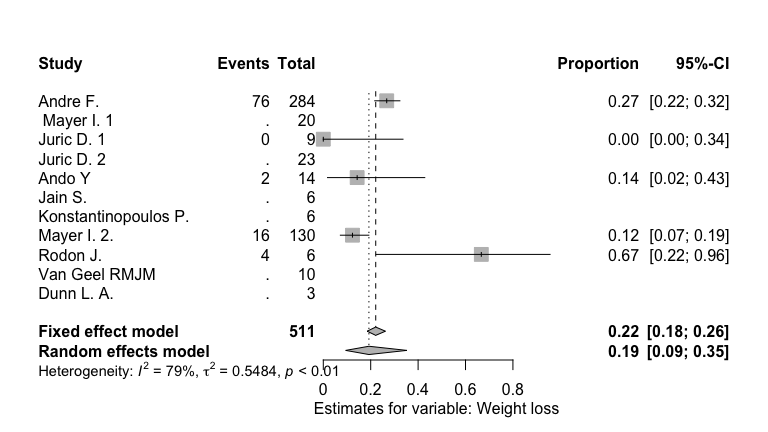

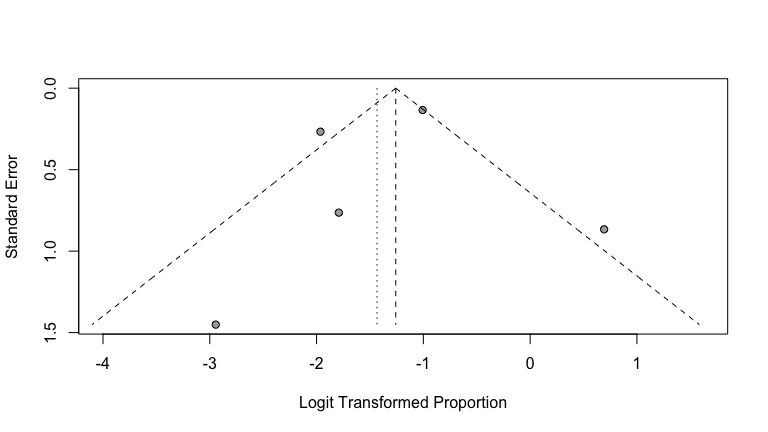


## Egger’s test
## Linear regression test of funnel plot asymmetry
##
## data: m11
## t = -0.39304, df = 3, p-value = 0.7206
## alternative hypothesis: asymmetry in funnel plot
## sample estimates:
## bias se.bias slope
## -0.6188449 1.5744923 -1.0687823


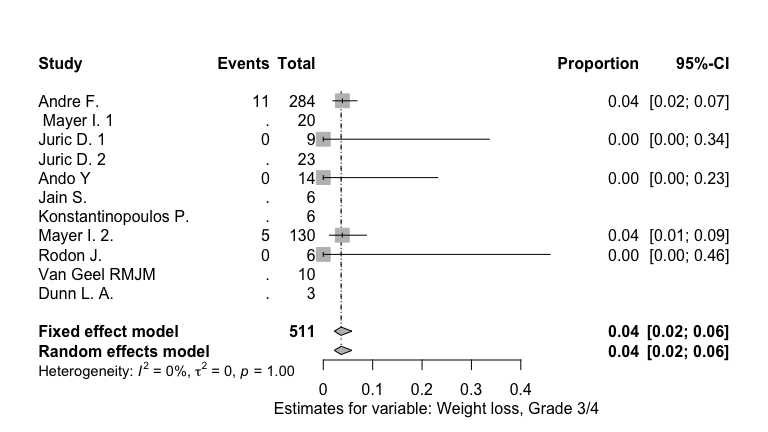

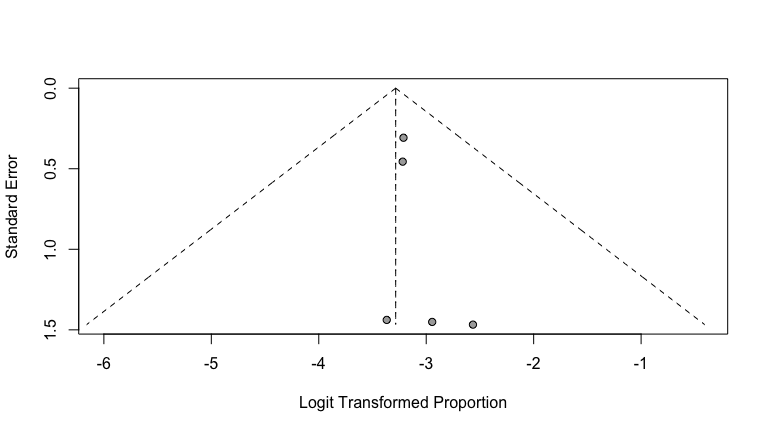


## Egger’s test
## Linear regression test of funnel plot asymmetry
##
## data: m12
## t = 1.229, df = 3, p-value = 0.3067
## alternative hypothesis: asymmetry in funnel plot
## sample estimates:
## bias se.bias slope
## 0.2183653 0.1776841 -3.2903195


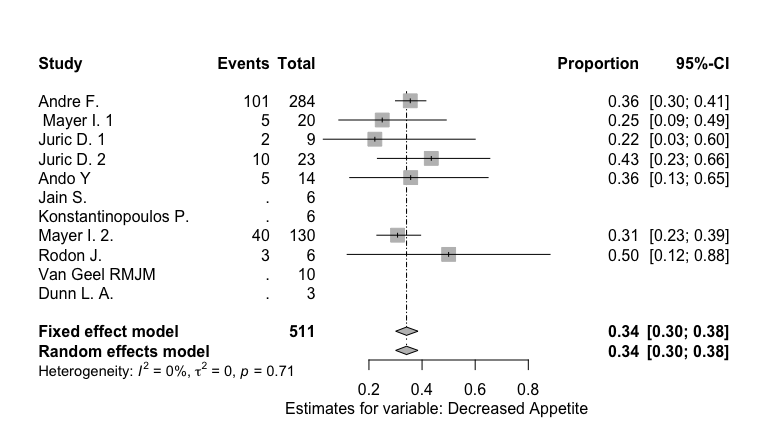

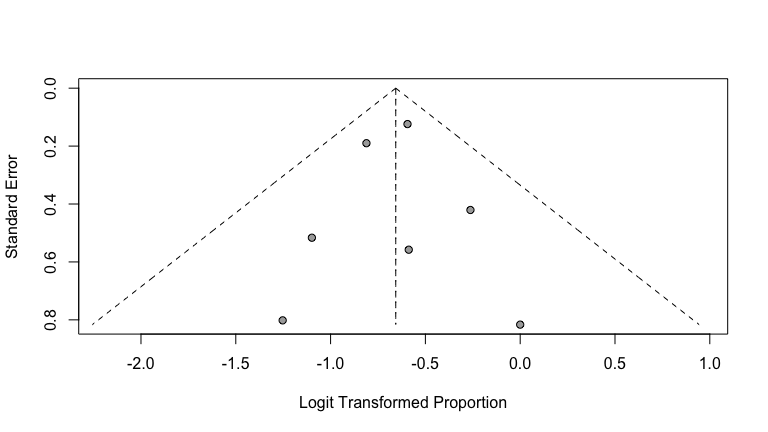


## Egger’s test
## Linear regression test of funnel plot asymmetry
##
## data: m13
## t = -0.12369, df = 5, p-value = 0.9064
## alternative hypothesis: asymmetry in funnel plot
## sample estimates:
## bias se.bias slope
## -0.06652256 0.53780808 -0.63738868


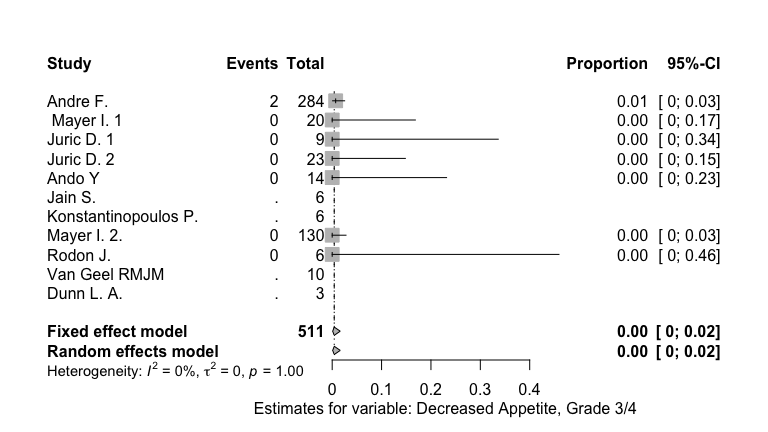

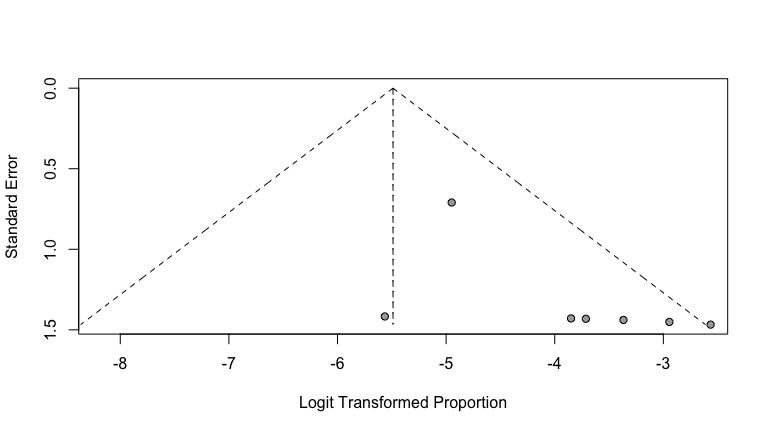


## Egger’s test
## Linear regression test of funnel plot asymmetry
##
## data: m14
## t = 1.9986, df = 5, p-value = 0.1021
## alternative hypothesis: asymmetry in funnel plot
## sample estimates:
## bias se.bias slope
## 1.793224 0.897225 -6.248308


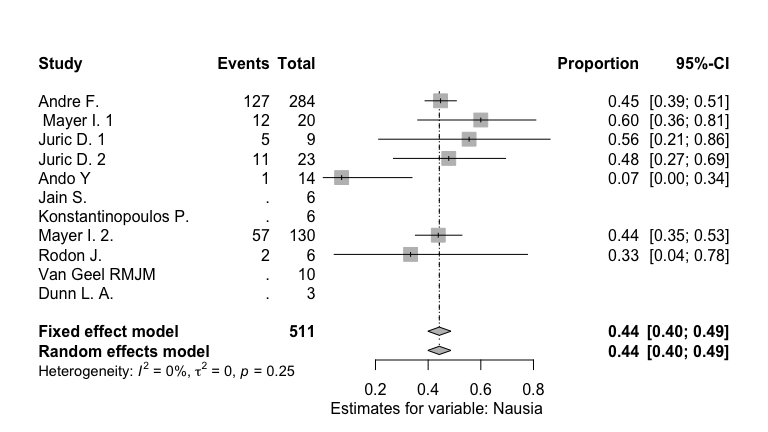

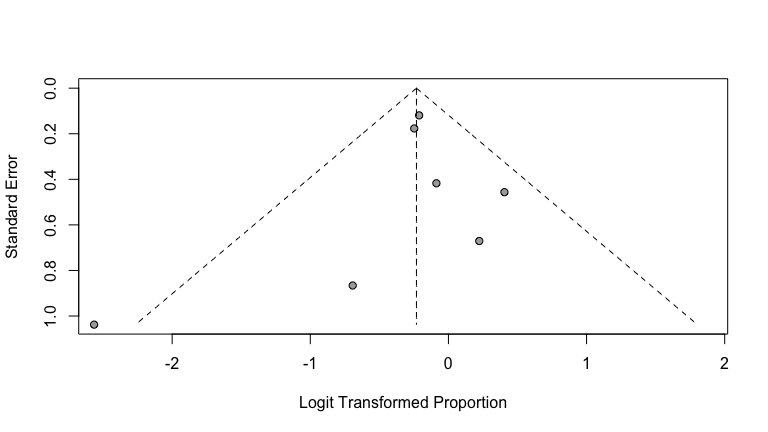


## Egger’s test
## Linear regression test of funnel plot asymmetry
##
## data: m15
## t = -0.41542, df = 5, p-value = 0.6951
## alternative hypothesis: asymmetry in funnel plot
## sample estimates:
## bias se.bias slope
## -0.3062444 0.7371918 -0.1480899


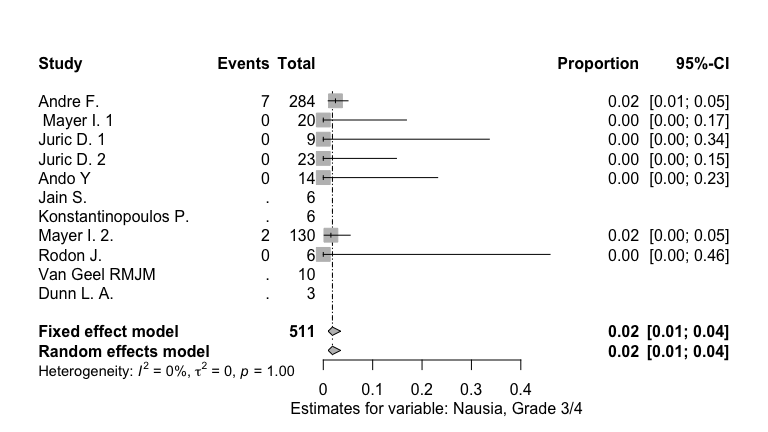

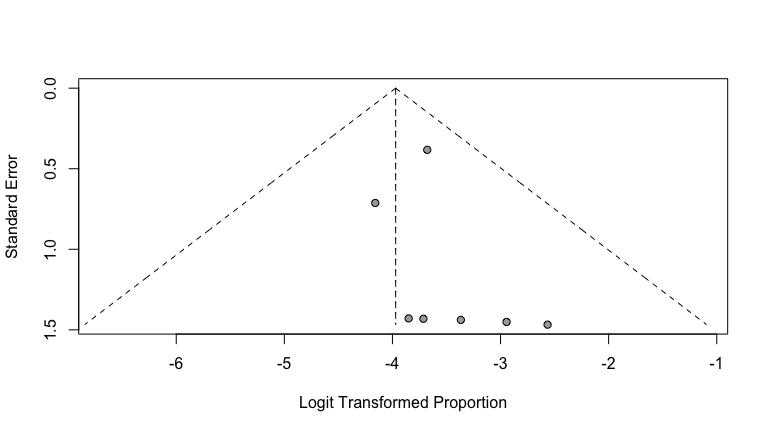


## Egger’s test
## Linear regression test of funnel plot asymmetry
##
## data: m16
## t = 1.0208, df = 5, p-value = 0.3542
## alternative hypothesis: asymmetry in funnel plot
## sample estimates:
## bias se.bias slope
## 0.3407006 0.3337629 -3.9086448


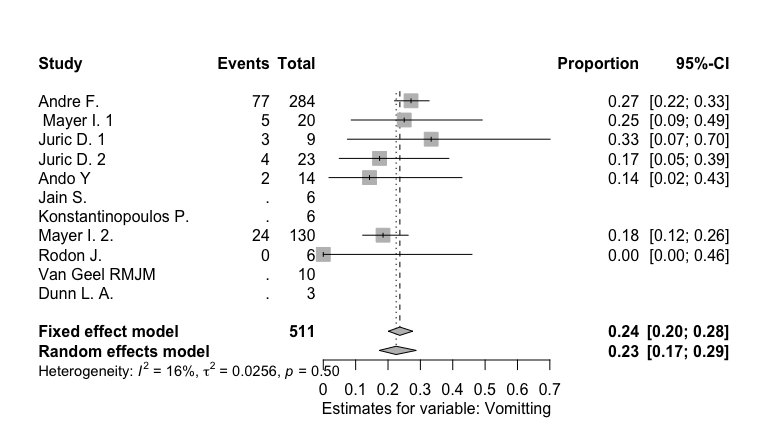

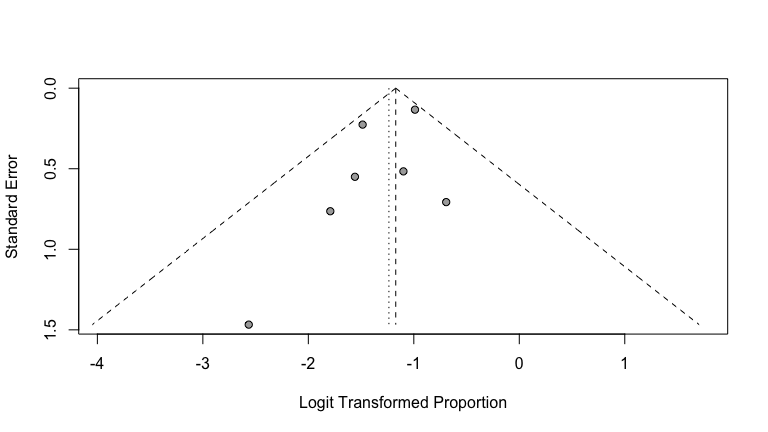


## Egger’s test
## Linear regression test of funnel plot asymmetry
##
## data: m17
## t = -1.3599, df = 5, p-value = 0.232
## alternative hypothesis: asymmetry in funnel plot
## sample estimates:
## bias se.bias slope
## -0.7754688 0.5702379 -0.9743348


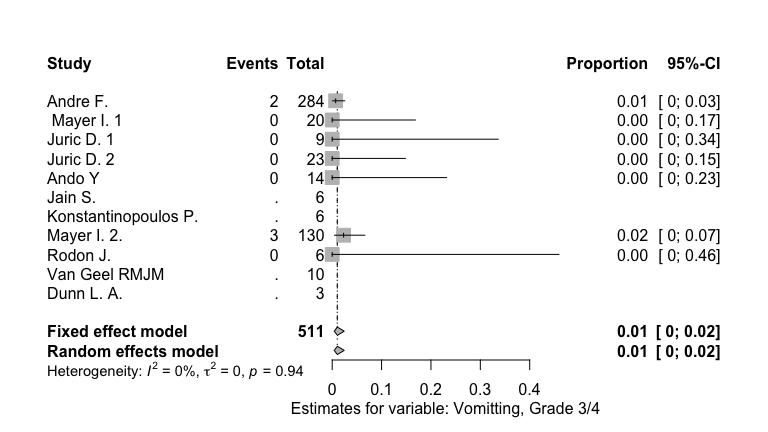

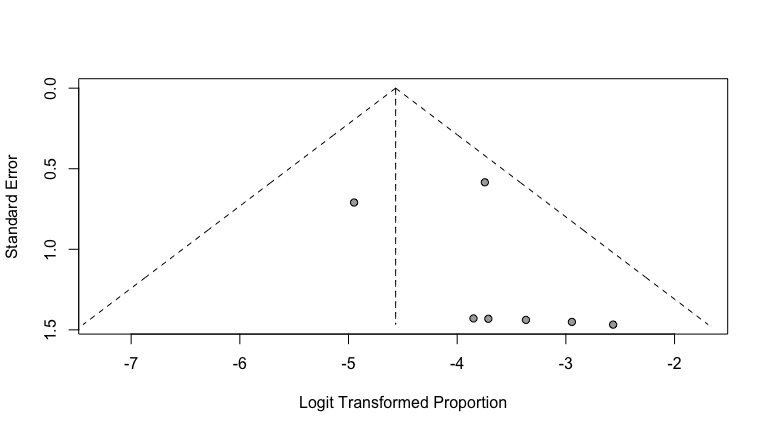


## Egger’s test
## Linear regression test of funnel plot asymmetry
##
## data: m18
## t = 1.4051, df = 5, p-value = 0.219
## alternative hypothesis: asymmetry in funnel plot
## sample estimates:
## bias se.bias slope
## 0.9834028 0.6998741 -4.8101537


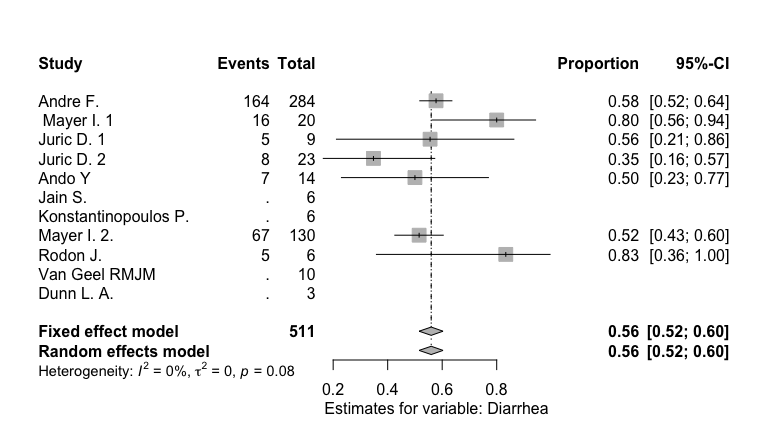

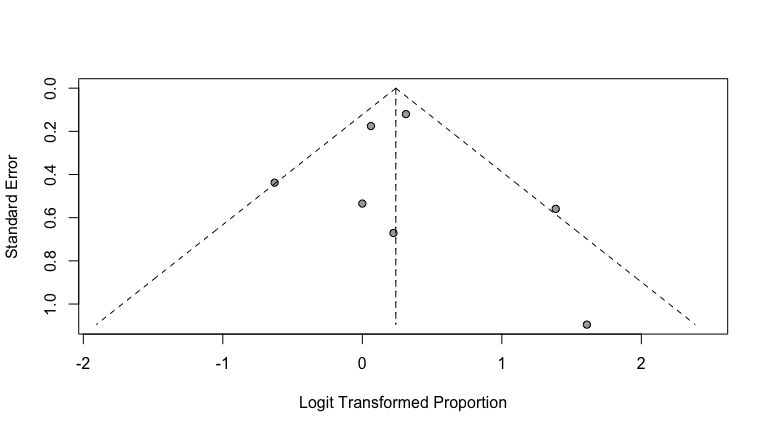


## Egger’s test
## Linear regression test of funnel plot asymmetry
##
## data: m19
## t = 0.28197, df = 5, p-value = 0.7893
## alternative hypothesis: asymmetry in funnel plot
## sample estimates:
## bias se.bias slope
## 0.2551714 0.9049475 0.1792931


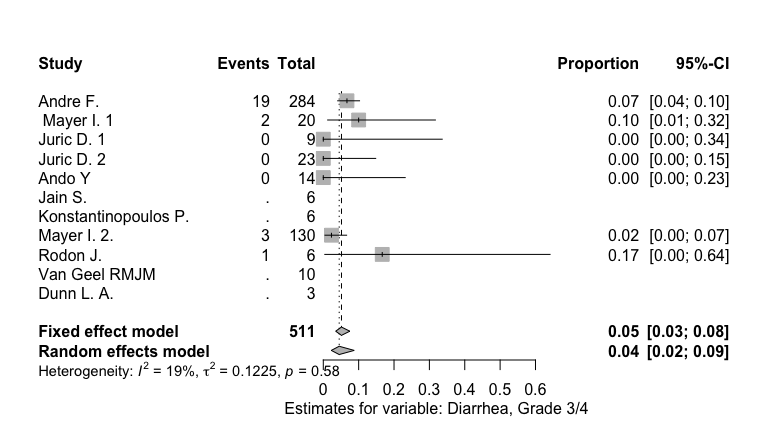

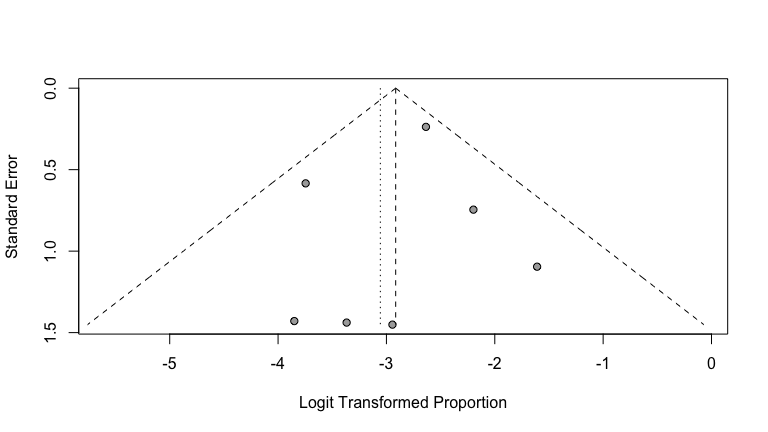


## Egger’s test
## Linear regression test of funnel plot asymmetry
##
## data: m20
## t = -0.48366, df = 5, p-value = 0.6491
## alternative hypothesis: asymmetry in funnel plot
## sample estimates:
## bias se.bias slope
## -0.3010875 0.6225236 -2.6197904


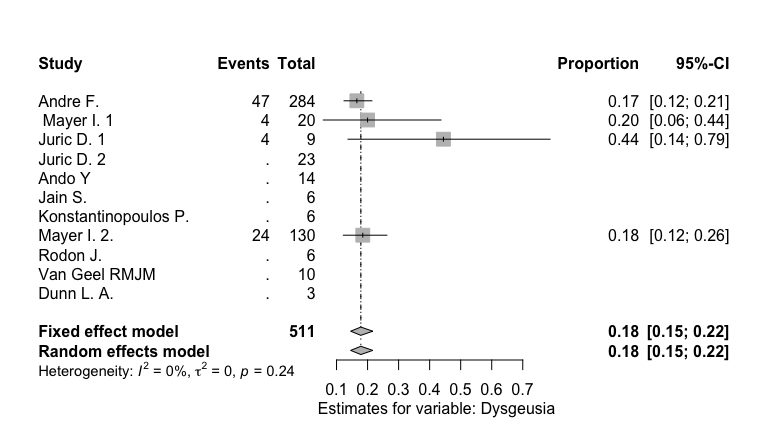

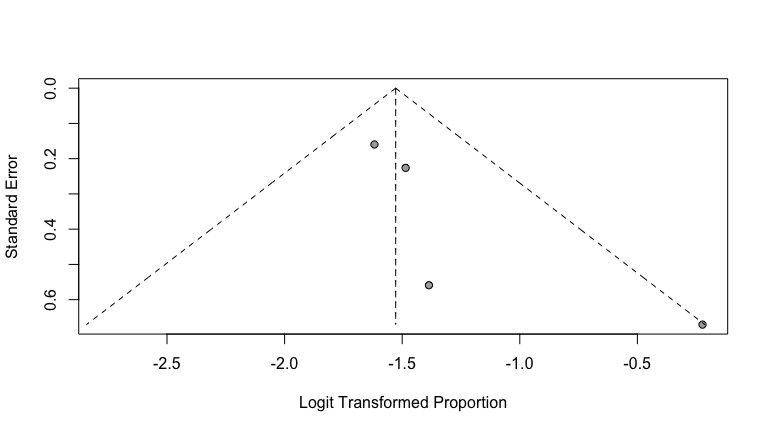


## Egger’s test
## Linear regression test of funnel plot asymmetry
##
## data: m21
## t = 2.1581, df = 2, p-value = 0.1636
## alternative hypothesis: asymmetry in funnel plot
## sample estimates:
## bias se.bias slope
## 1.7459259 0.8090116 -1.8974419


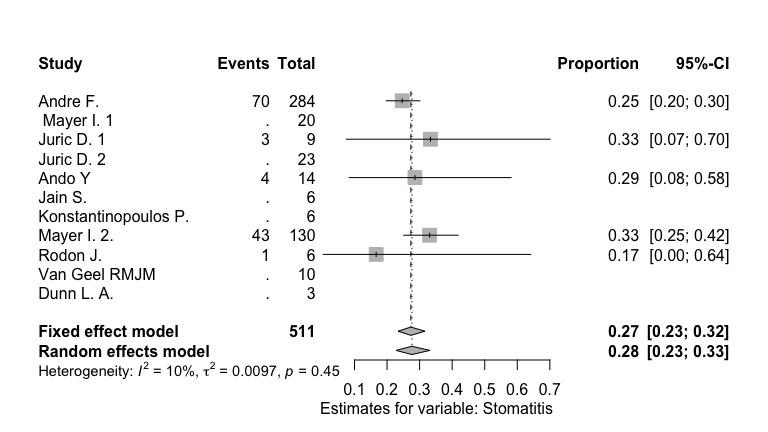

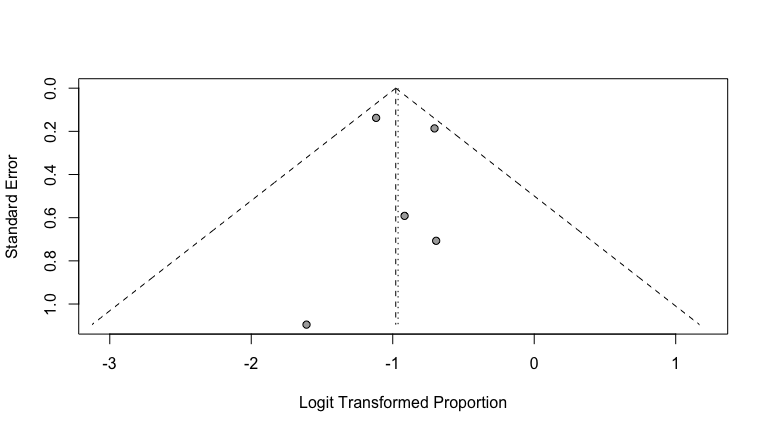


## Egger’s test
## Linear regression test of funnel plot asymmetry
##
## data: m23
## t = 0.16466, df = 3, p-value = 0.8797
## alternative hypothesis: asymmetry in funnel plot
## sample estimates:
## bias se.bias slope
## 0.1343560 0.8159385 -0.9953032


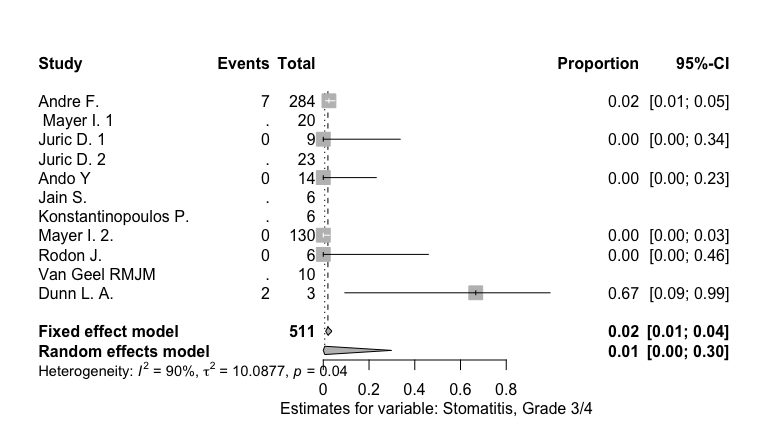

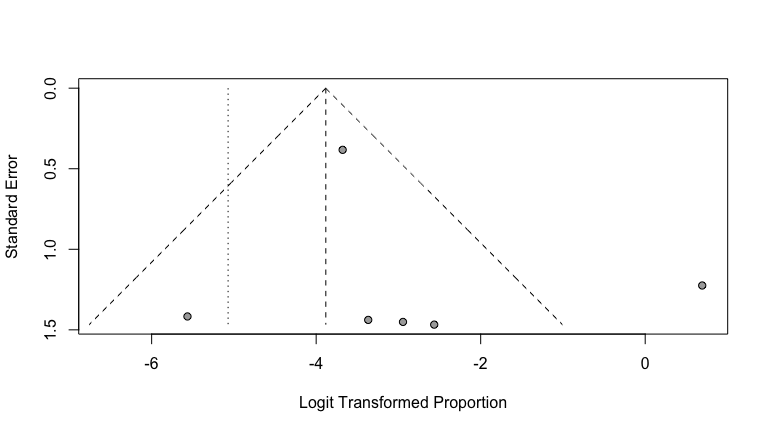


## Egger’s test
## Linear regression test of funnel plot asymmetry
##
## data: m24
## t = 0.70503, df = 4, p-value = 0.5197
## alternative hypothesis: asymmetry in funnel plot
## sample estimates:
## bias se.bias slope
## 0.9127324 1.2945961 -3.9616759


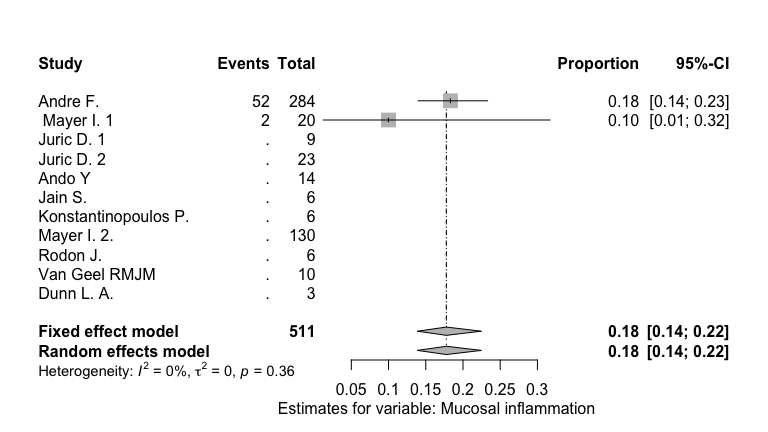

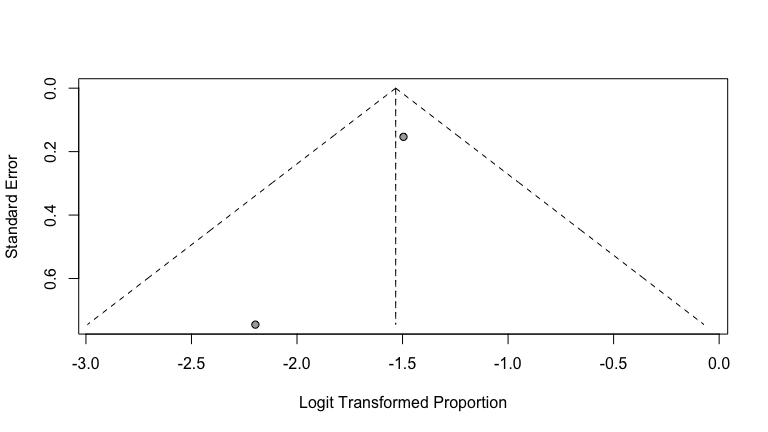

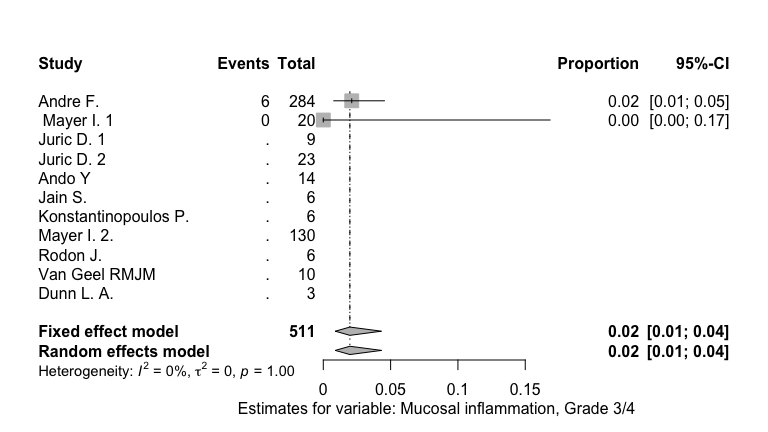

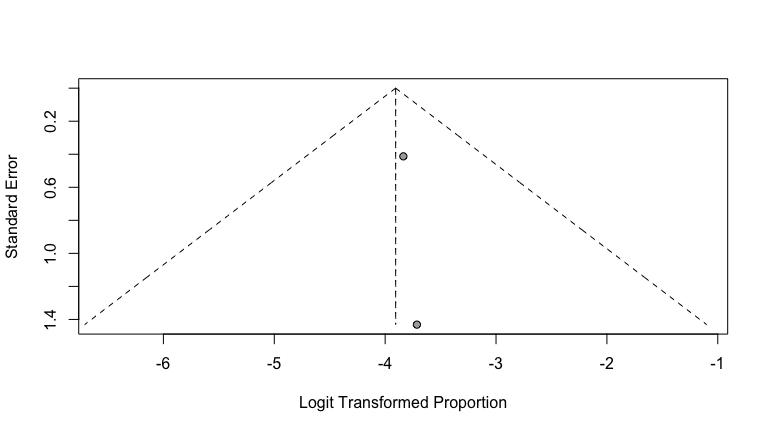

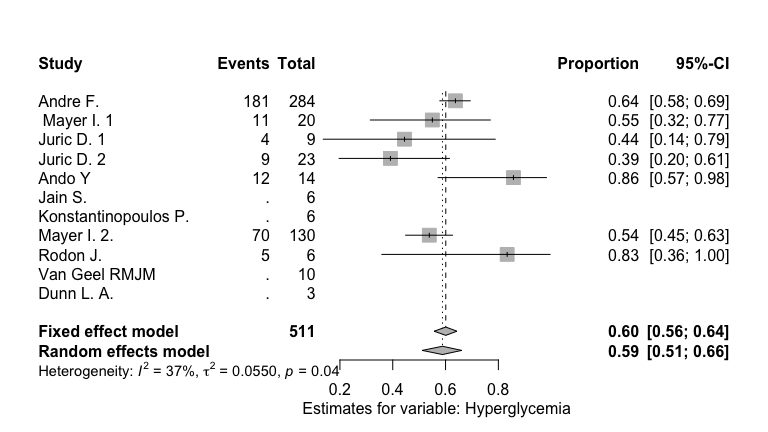

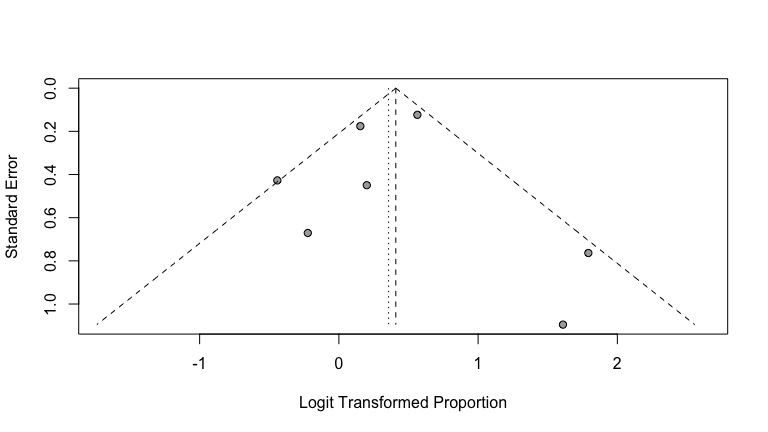


## Egger’s test
## Linear regression test of funnel plot asymmetry
##
## data: m27
## t = -0.12561, df = 5, p-value = 0.9049
## alternative hypothesis: asymmetry in funnel plot
## sample estimates:
## bias se.bias slope
## -0.1240245 0.9873456 0.4172750


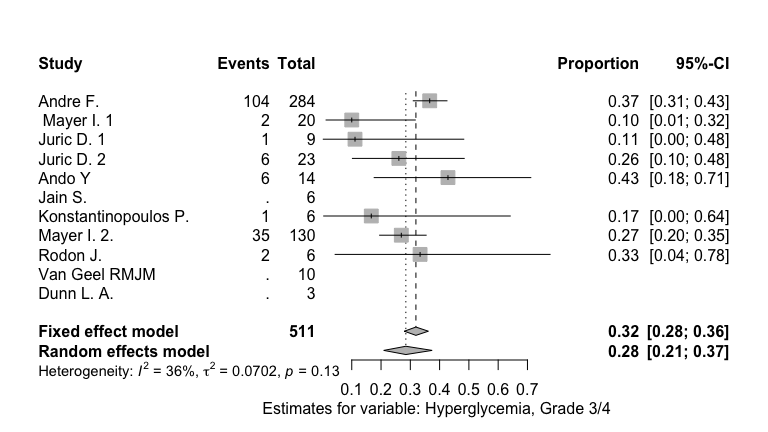

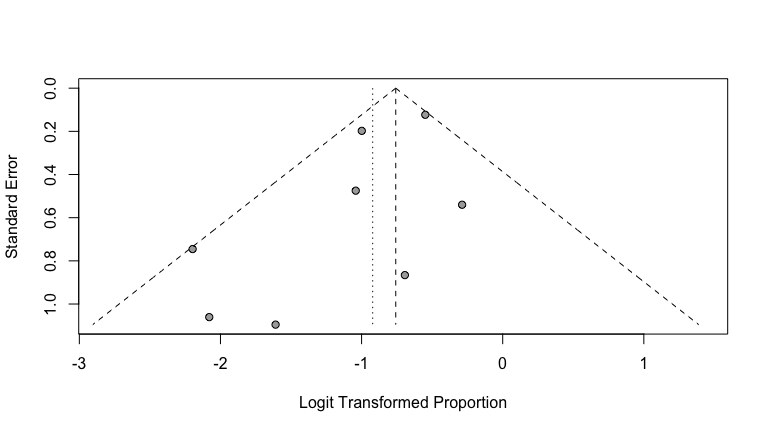


## Egger’s test
## Linear regression test of funnel plot asymmetry
##
## data: m28
## t = -1.887, df = 6, p-value = 0.1081
## alternative hypothesis: asymmetry in funnel plot
## sample estimates:
## bias se.bias slope
## -1.0833307 0.5741048 -0.4999060


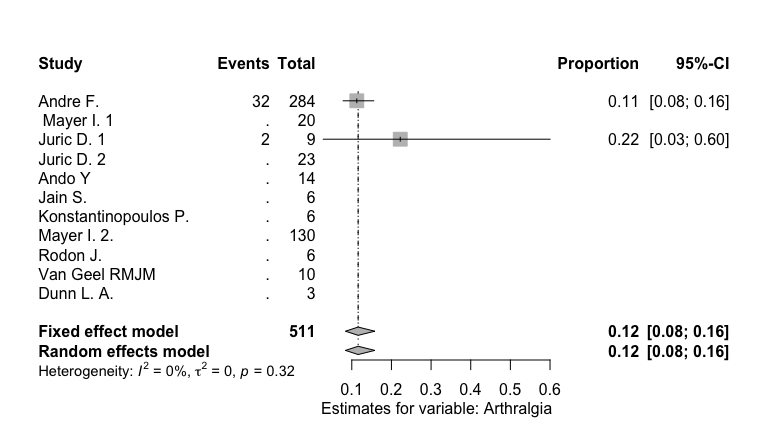

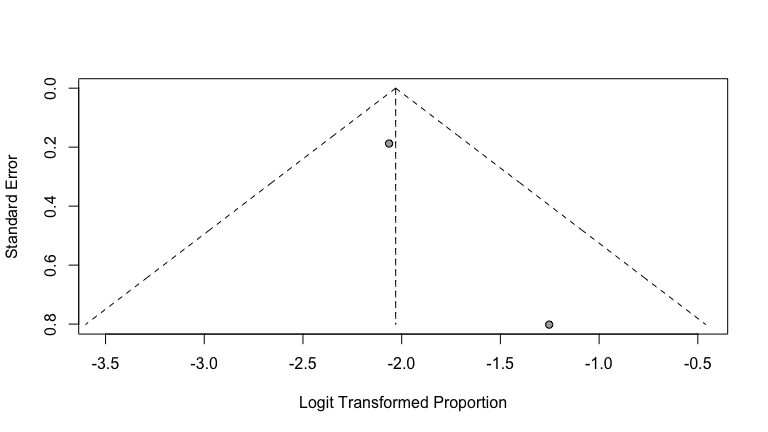

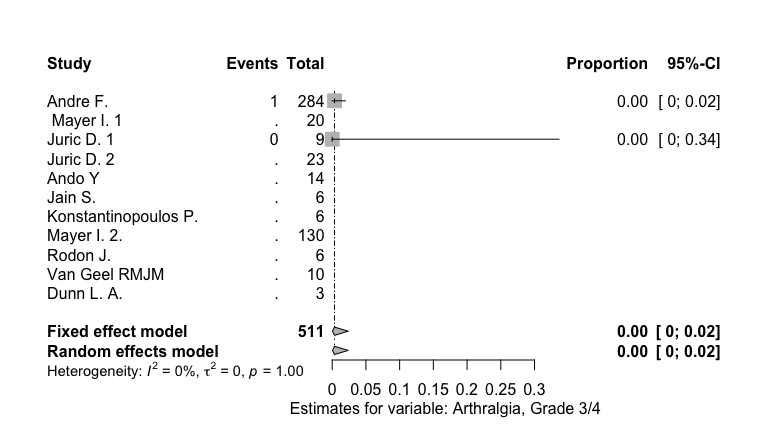

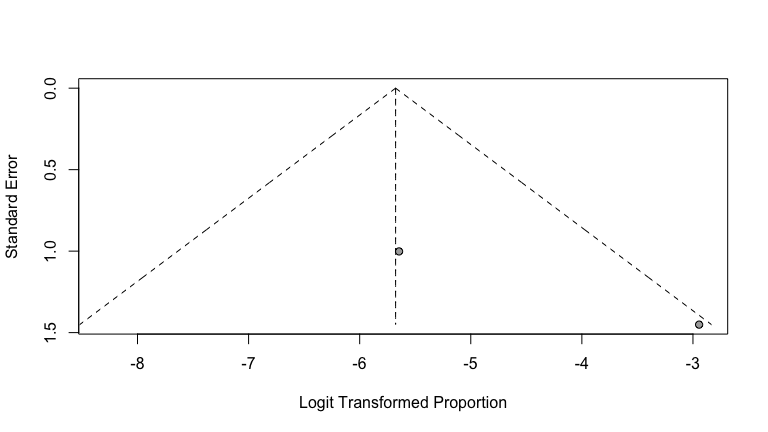

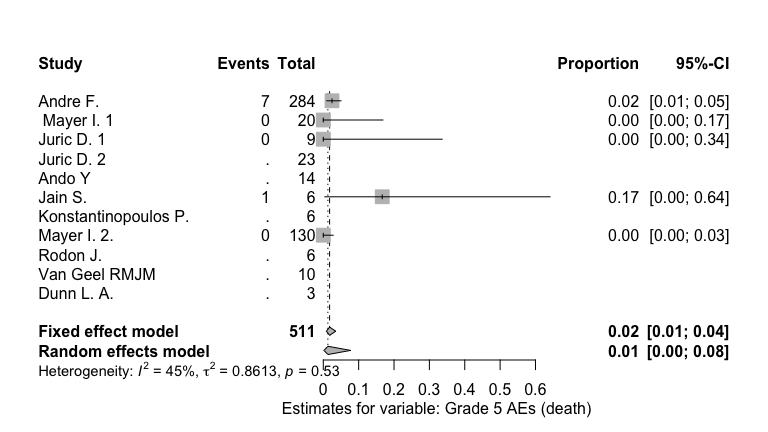

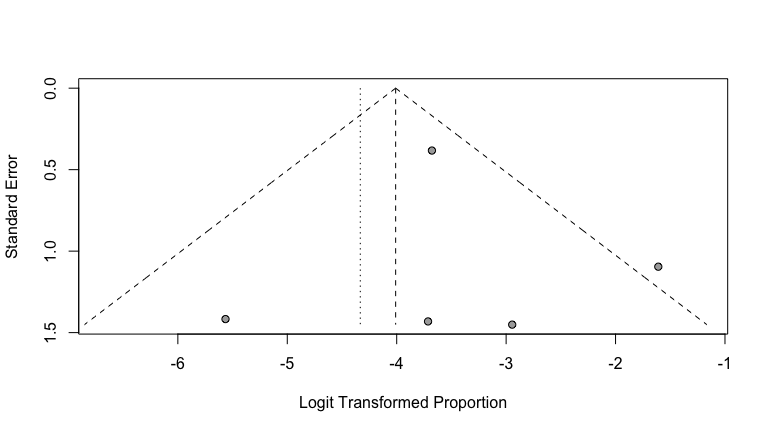


## Egger’s test
## Linear regression test of funnel plot asymmetry
##
## data: m31
## t = 0.21123, df = 3, p-value = 0.8462
## alternative hypothesis: asymmetry in funnel plot
## sample estimates:
## bias se.bias slope
## 0.2280402 1.0795779 -3.6963034


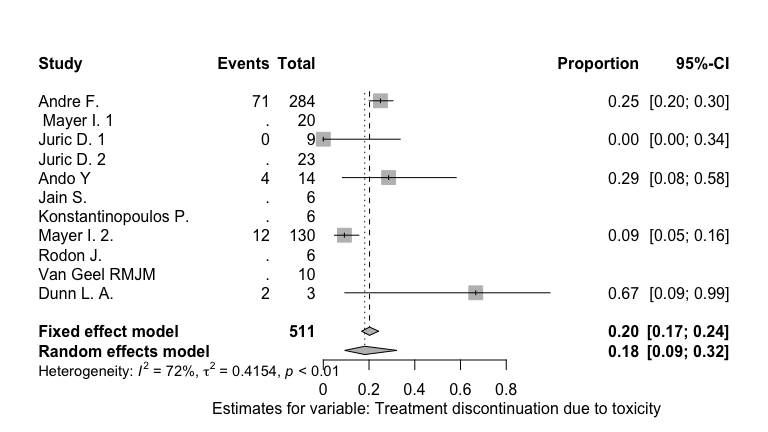

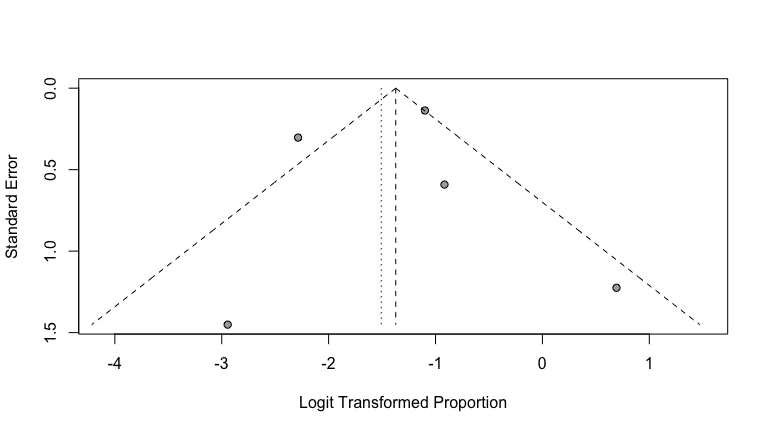


## Egger’s test
## Linear regression test of funnel plot asymmetry
##
## data: m32
## t = -0.27762, df = 3, p-value = 0.7993
## alternative hypothesis: asymmetry in funnel plot
## sample estimates:
## bias se.bias slope
## -0.4399187 1.5846282 -1.1870207
